# The Efficacy and Safety of Ultrasound-Guided, Bi-Level, Erector Spinae Plane Block With Different Doses of Dexmedetomidine for Patients Undergoing Video-Assisted Thoracic Surgery: A Randomized Controlled Trial

**DOI:** 10.3389/fmed.2021.577885

**Published:** 2021-11-25

**Authors:** Xiujuan Gao, Tonghang Zhao, Guangjun Xu, Chunguang Ren, Guoying Liu, Ke Du

**Affiliations:** ^1^Department of Anesthesiology, Liaocheng People's Hospital, Liaocheng, China; ^2^Department of Thoracic Surgery, Liaocheng People's Hospital, Liaocheng, China

**Keywords:** erector spinae plane block, video-assisted thoracic surgery, ultrasound, dexmedetomidine, ropivacaine

## Abstract

**Background:** The anesthetic characteristics of ultrasound-guided bi-level erector spinae plane block (ESPB) plus dexmedetomidine (Dex) remain unclear. We compared the efficacy and safety of ultrasound-guided bi-level ESPB plus different doses of Dex in patients undergoing video-assisted thoracic surgery (VATS).

**Methods:** One-hundred eight patients undergoing VATS were randomized into three groups: R group (*n* = 38, 15 ml of 0.375% ropivacaine with 0.1 mg/kg dexamethasone), RD1 group (*n* = 38, 15 ml of 0.375% ropivacaine plus 0.5 μg/kg DEX with 0.1 mg/kg dexamethasone) and RD2 group (*n* = 38, 15 ml of 0.375% ropivacaine plus 1.0 μg/kg DEX with 0.1 mg/kg dexamethasone). The primary outcome was the pain 12 h after surgery. Secondary outcomes included the Prince Henry Hospital Pain Score; hemodynamics; consumption of sufentanil; anesthetized dermatomal distribution; recovery time; rescue analgesia; satisfaction scores of patients and surgeon; quick recovery index; adverse effects; the prevalence of chronic pain and quality of recovery.

**Results:** The visual analog scale (VAS) and the Prince Henry pain score were significantly lower in both the RD1 and RD2 groups during the first 24 h after surgery (*P* < 0.05). Both VAS with coughing and the Prince Henry pain score were significantly lower in the RD2 group than in the RD1 group 8–24 h after surgery (*P* < 0.05). Both heart rate and mean arterial pressure were significantly different from T2 to T6 in the RD1 and RD2 groups (*P* < 0.05). The receipt of remifentanil, propofol, Dex, and recovery time was significantly reduced in the RD2 group (*P* < 0.05). The requirement for sufentanil during the 8–72 h after surgery, less rescue medication, and total press times were significantly lower in the RD2 group (*P* < 0.05). The time to the first dose of rescue ketorolac was significantly longer in the RD2 group (*P* < 0.05). Further, anal exhaust, removal of chest tubes, and ambulation were significantly shorter in the RD2 group (*P* < 0.05). The incidence of tachycardia, post-operative nausea and vomiting, and chronic pain was significantly reduced in the RD2 group, while the QoR-40 score was significantly higher in the RD2 group (*P* < 0.05).

**Conclusions:** Pre-operative bi-level, single-injection ESPB plus 1 μg/kg DEX provided superior pain relief and long-term post-operative recovery for patients undergoing VATS.

**Clinical Trial Registration:** http://www.chictr.org.cn/searchproj.aspx.

## Introduction

Guidelines for enhanced recovery after thoracic surgery (ERATS) aimed at early mobilization and minimal perioperative opioid use without increasing hospital readmission or mortality have been published recently. Video-assisted thoracic surgery is one of the most minimally invasive techniques that allow faster recovery after thoracic surgery in this guideline (1). However, the optimal multimodal analgesia regimen for video-assisted thoracic surgery (VATS) has not been well-established (2). The poor management of post-operative pain after VATS may result in pulmonary complications, increase chronic pain and the duration of hospital stay, and subsequently, affect the quality of life of patients (3, 4). Previous study also reported that the most important risk factor for chronic pain after VATS was the occurrence of moderate-to-severe post-operative pain (5).

Regional anesthesia blocks should be considered as a vital component of the multimodal analgesia regimen. These techniques can decrease complications of opioid use, reduce the length of hospital stay, and improve the efficiency of healthcare resources (6). In recent decades, thoracic epidural block (TEB) and paravertebral block (PVB) have been successfully used for analgesia in thoracic surgery (7). Thoracic epidural block is considered the gold standard technique for pain control after thoracotomy, however, it is not compatible with the concept of ERATS owing to technical complexities and significant potential complications (8). Besides, PVB can cause pneumothorax and total spinal anesthesia (9).

Ultrasound-guided regional anesthesia allows the real-time visualization of anatomical structures, needle advancement, and local anesthetic (LA) spread. Ultrasound-guided thoracic fascial plane blocks, which are relatively simple and safe to perform, can provide alternative analgesic options (10). Ultrasound-guided erector spinae plane block (ESPB), a novel interfacial plane block first described in 2016, has recently been applied in clinical practice as it is less invasive, however, the exact mechanism and its injectate spread are still controversial (11–13). Recent studies have shown that ESPB is a potentially promising effective and safer alternative to TEB and PVB without significant complications (14, 15). Local anesthetic combined with dexmedetomidine (DEX) has been reported to prolong analgesia in TEB, PVB, brachial plexus blocks, and serratus anterior plane block (SAPB) (16). However, it is presently unclear whether adjuncts such as DEX will significantly prolong the duration of ESPB in the same manner. We hypothesized that ultrasound-guided, bi-level ESPB plus different doses of dexmedetomidine (DEX) could improve post-operative analgesia and post-operative recovery in patients undergoing VATS.

## Methods

### Patients

The institutional review board of the Liaocheng People's Hospital provided ethics approval to conduct the trial (No. 2014001); this trial is an extension of our initial protocol which has been registered at chictr.org (ChiCTR-TRC-14004191 and ChiCTR-IPR-15007229). Written informed consent was obtained from patients or their guardians. Patients undergoing VATS at the Liaocheng People's Hospital between May 2018 and November 2019 were recruited. Eligible patients were those between 45 and 65 years old, scheduled for lobectomy under complete VATS (17), and had an American Society of Anesthesiologists (ASA) status of I or II. Patients were excluded if they had clinically serious cardiovascular or peptic ulcer diseases; history of allergy to LA, DEX, or non-steroidal anti-inflammatory drugs (NSAIDs); body mass index (BMI) >30 kg/m^2^; infection at the puncture site; history of chronic pain or analgesic use for nearly 6 months; diabetes; unable to use the visual analog scale (VAS) or patient-controlled intravenous analgesia (PCIA) system.

### Randomization and Blinding

Randomization was performed using a computer-generated randomization table. The anesthetic nurses in the acute pain services (APS) instructed the patients on how to use a 10-cm VAS scale (0 = no pain, 10 = maximum pain imaginable) and PCIA system and performed all the post-operative assessments. Another anesthetic nurse who did not participate in the study prepared the drugs for ESPB and PCIA according to the randomization table. The patients, anesthesiologists, anesthetic nurses, and the surgeon were all blinded to this study. One-hundred eight patients undergoing VATS were randomized into three groups: R group (*n* = 36, 30 ml 0.375% ropivacaine with 0.1 mg/kg dexamethasone), RD1 group (*n* = 36, 30 ml 0.375% ropivacaine with 0.1 mg/kg dexamethasone plus 0.5 μg/kg DEX), and RD2 group (*n* = 36, 30 ml of 0.375% ropivacaine with 0.1 mg/kg dexamethasone plus 1.0 μg/kg DEX).

### Ultrasound-Guided Bi-Level ESPB

Erector spinae plane block was performed as described in a previous study (18). Briefly, a unilateral ultrasound-guided ESPB (SonoSite, Washington, USA) was administered under aseptic conditions by the same experienced anesthesiologist in the anesthesia preparation room. The patients were under standardized monitoring with 2–3 L/min oxygen through a nasal cannula. Then, 2 mg midazolam and 5 μg sufentanil were administered intravenously before the patients were placed in a lateral position. The T4 spinous process was identified by palpation starting from C7 downward; then, a high-frequency probe was placed 2–3 cm lateral to the T4 transverse process longitudinally. After visualizing the trapezius, rhomboid major, erector spinae muscles, and transverse processes, an in-plane approach was adopted. An 8 cm, 22-gauge needle (Stimuplex Ultra 360; B. Braun, Melsungen, Germany) was inserted in the cephalad-to-caudad direction with a shallow trajectory (30–40°) in the fascial plane, deep to the erector spinae muscle. Afterward, 2 ml saline was injected to confirm the proper injection site and 15 ml 0.375% ropivacaine with 0.1 mg/kg dexamethasone and different doses of DEX were then injected. The same procedure was performed at the T6 transverse process level. The range of the dermatomal distribution was tested by the pinprick method 30 min after the block.

### Anesthesia Management

No pre-operative medication and the same multimodal analgesia regimen were used in all patients. To facilitate the endotracheal intubation, 1.5–2.5 mg/kg propofol, 0.2 μg/kg sufentanil, 0.2 mg/kg cisatracurium, and 1 mg/kg lidocaine were used. The position of the tube was verified using fiberoptic bronchoscopy. Next, 1 mg/kg flurbiprofen axetil was administered intravenously for pre-emptive analgesia and 0.1 mg/kg dexamethasone was administered for the prophylaxis of post-operative nausea and vomiting (PONV) before the surgical incision. We adopted the total intravenous anesthesia (TIVA) technique during the surgery to decrease the risk of PONV (19). Target controlled infusion (TCI) 2–4 μg/ml propofol, 0.2–0.7 μg/kg/h Dex, and 0.1–0.2 μg/kg/min remifentanil administration was adjusted to target a bispectral index (BIS) between 40 and 60. Further, 0.1 mg/kg cisatracurium was infused as necessary to help maintain one to two twitches in response to the train-of-four (TOF) stimulus of the ulnar nerve. One-lung mechanical ventilation was set with a tidal volume of 4–6 ml/kg and a peak airway pressure of <25 cm H_2_O according to the protective lung ventilation strategy (20). The neuromuscular blockade was antagonized by 0.01 mg/kg atropine and 0.02 mg/kg neostigmine because residual paralysis was associated with a higher risk of post-operative pulmonary complications. Additionally, 5 mg of intravenous tropisetron was given at the end of the surgery for PONV prophylaxis. All VATS procedures were performed by the same surgeon.

### Post-operative Pain Management

All the patients were extubated at the end of surgery and transferred to the post-anesthesia care unit (PACU). Patient-controlled intravenous analgesia was programmed to deliver 0.02 μg/kg/h sufentanil and 0.02 μg/kg sufentanil bolus, followed by a 15-min lockout period. Post-operative analgesia with 1 g of intravenous acetaminophen was provided every 8 h; a 30 mg rescue dose of intravenous ketorolac if the VAS at rest is >3 or according to the demands of the patients; 4 μg of sufentanil was administered if the VAS at rest was still >3 after 30 min. Hypotension was defined as systolic arterial pressure (SAP) <90 mmHg or a decrease of >20% compared with the baseline and was treated with 6 mg of intravenous ephedrine. Tachycardia was defined as heart rate increased by >20% compared with the baseline and was treated with 10 mg of intravenous esmolol.

### Measurements

The primary outcome was VAS both at rest and with coughing during the 12 h after surgery. The secondary outcomes included the Prince Henry Hospital Pain Score (0 = no pain on coughing, 1 = pain on coughing, but not on deep breathing, 2 = pain on deep breathing but not at rest, 3 = slight pain at rest, 4 = severe pain at rest) (21); requirement of sufentanil (recorded at 1, 4, 8, 12, 24, 48, and 72 h post-operatively); hemodynamics [recorded at the following time points: arrival at the operating room (T0), before intubation (T1), after intubation (T2), before incision (T3), 30 min after one-lung ventilation (T4), at extubation (T5), and 1 (T6), 4 (T7), 8 (T8), and 12 (T9) h post-operatively]; anesthetized dermatomal distribution; PACU recovery time; total number of PCIA presses; number of patients needing rescue analgesia; time to first rescue analgesic; satisfaction scores of patients and surgeon (assessed using an 11-point Likert scale: 0 = entirely unsatisfied, 10 = fully satisfied); time of feeding, ambulation, exhaust, and removal of chest tubes; adverse effects; and length of hospital stay. Additionally, the prevalence of chronic pain and the quality of recovery [40-item QoR questionnaire (QoR-40): scores ranging from 40 to 200, representing very poor to excellent] were assessed 3 months after surgery (22).

### Statistical Analysis

Our sample size was calculated based on VAS with coughing 12 h post-operatively. According to our preliminary study, VAS with coughing 12 h post-operatively was 3.2 ± 0.7. Assuming an alpha value of 0.05 and a beta value of 0.2 for a 1.0-point difference, the calculated sample size was 32 patients per group. Considering the dropout rate, we enrolled a total of 108 patients in this study.

Statistical analysis was performed with SPSS for Windows Version 22.0 (IBM, Ney York, USA). The Kolmogorov–Smirnov test was used to assess the distribution of variables. The homogeneity of variance was determined using Levene tests. Normally distributed data were expressed as mean SD, non-normally distributed data were expressed using median (interquartile range), and categorical data were expressed as number (*n*) and percentage (%). Repeated-measures two-way ANOVA was used to evaluate the differences at different time points among the groups. Bonferroni multiple comparisons were performed for multiple comparisons. The non-normally distributed data were analyzed with the Kruskal–Wallis test, chi-square tests, or Fisher's exact tests. Probability (*P*) values <0.05 were considered statistically significant.

## Results

### Baseline Characteristics

**Figure 1:**
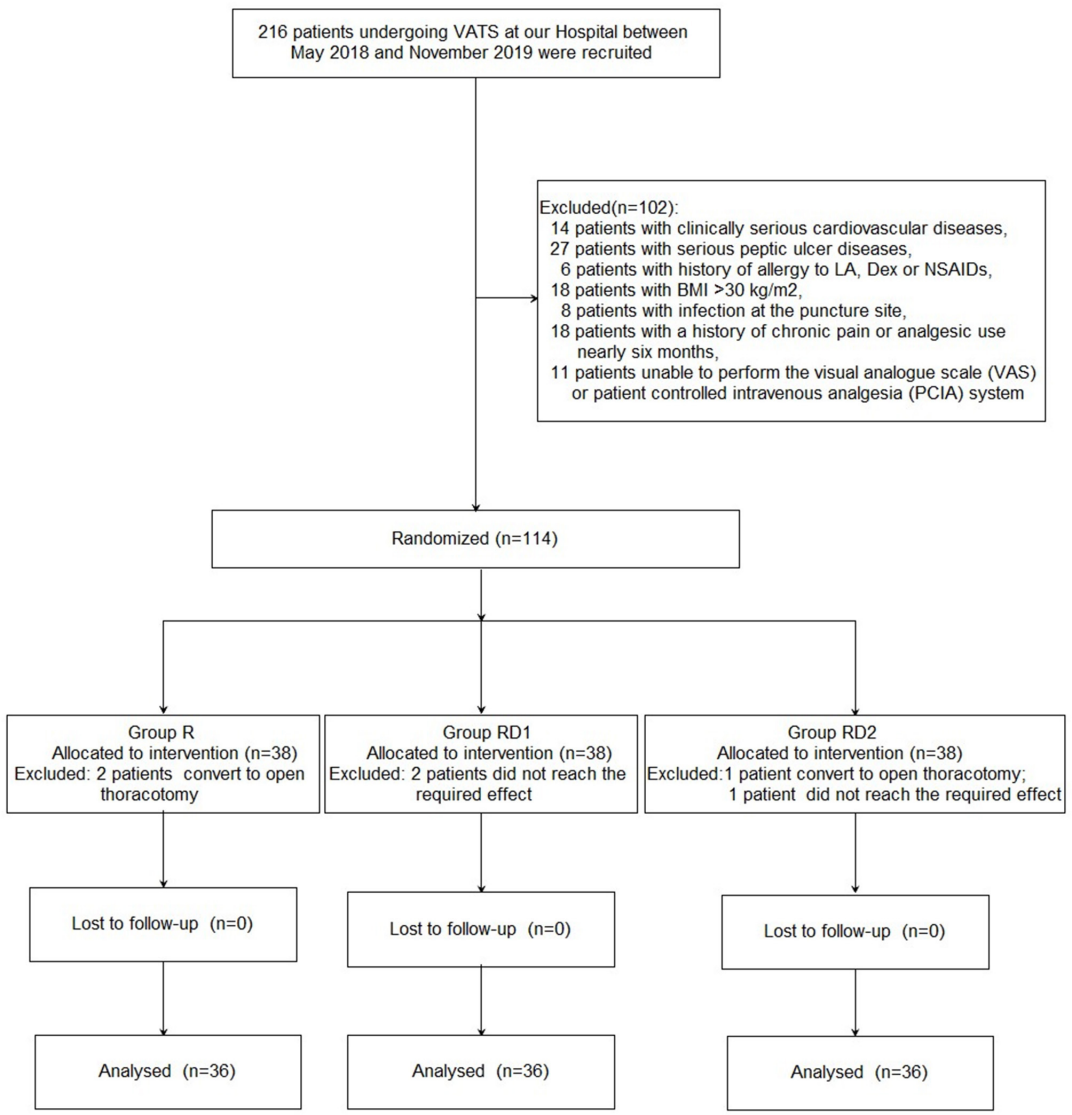
Patient consort flow diagram.

Figure 1 shows the details of patient enrollment according to the consolidated standards of reporting trials (CONSORT) guidelines. Two hundred sixteen patients undergoing VATS at our hospital between May 2018 and November 2019 were recruited. Of these, 102 patients were excluded for the following reasons: 14 patients with clinically serious cardiovascular diseases, 27 with serious peptic ulcers, six with a history of allergy to LA, Dex, or NSAIDs, 18 patients with BMI >30 kg/m^2^, eight with infection at the puncture site, 18 with a history of chronic pain or analgesic use for nearly 6 months, and 11 patients who were unable to use the VAS or PCIA system. Further, three patients were excluded because the sensory block did not reach the required level (two patients from the RD1 group, and one from the RD2 group), and three patients were excluded because of conversion to open thoracotomy (two patients from the R group, and one patient from the RD2 group). Finally, 108 patients were enrolled in this study (*n* = 36, each group). There were no significant differences with respect to age, sex, BMI, ASA grade, location, pulmonary function, or pre-operative comorbidities among the three groups (*P* > 0.05, Table 1).

**Table 1:** Demographic characteristics.

	**Group R (***n*** = 36)**	**Group RD1 (***n*** = 36)**	**Group RD2 (***n*** = 36)**	***P*** **-values**
Age (years)	57.23 ± 6.31	55.72 ± 4.67	56.56 ± 5.02	0.493
Sex (female/male, *n*)	16/20	13/23	15/21	0.765
BMI (kg·m^−2^)	22.39 ± 2.16	22.32 ± 1.90	22.47 ± 1.88	0.950
ASA I to II, n	11/25	9/27	10/26	0.871
Location (left/right), *n*	21/15	23/13	18/18	0.487
FEV1/FVC (%)	89.22 ± 3.35	87.85 ± 3.43	88.41 ± 3.62	0.246
Comorbidity, *n* (%)				0.956
Hypertension	11 (30.56%)	12 (33.33%)	13 (36.11%)	
Diabetes mellitus	8 (22.22%)	6 (16.67%)	9 (25.00%)	
Coronary heart disease	4 (11.11%)	3 (8.33 %)	6 (16.67%)	

*Variables presented as mean ± SD or number of patients n (%). BMI, body mass index; ASA, American Society of Anesthesiology; FEV1/FVC, Forced vital capacity rate of 1 s/Forced vital capacity*.

### Intraoperative Variables

**Figure 2:**
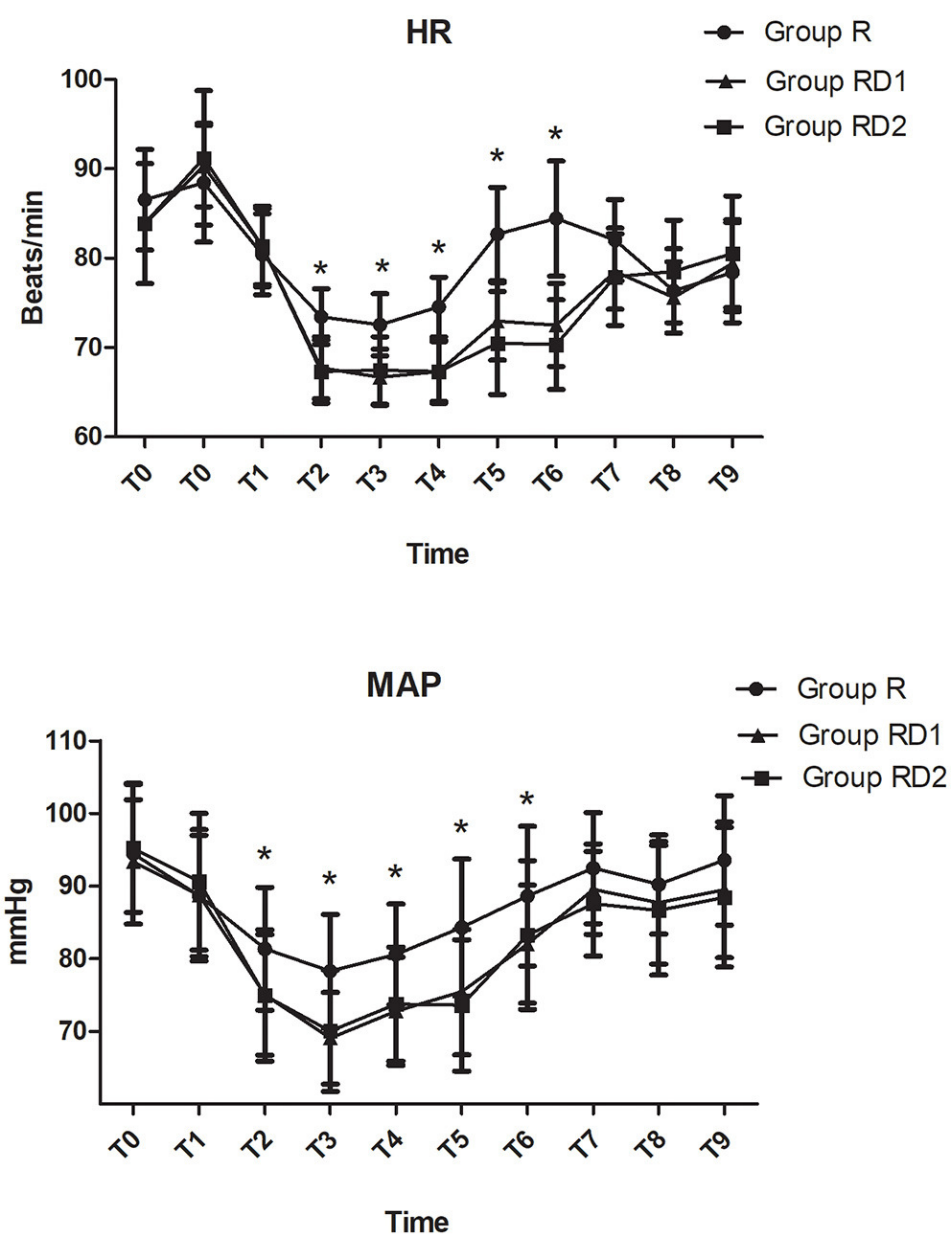
Intraoperative hemodynamic changes. T0, arrival at the operating room; T1, before intubation; T2, after intubation; T3, before incision; T4, 30 min after one-lung ventilation; T5, extubation; T6, 1 h post-operatively; T7, 4 h post-operatively; T8, 8 h post-operatively; T9, 12 h post-operatively. *P < 0.05 vs. group R.

Both heart rate and mean arterial pressure were significantly decreased from T2 to T6 in the RD1 and RD2 groups compared with the R group (*P* < 0.05, Figure 2). However, there were no significant differences between the RD1 and RD2 groups (*P* > 0.05, Figure 2). The requirement of propofol, remifentanil, and Dex was significantly lower in the RD1 and RD2 groups than in the R group (*P* < 0.05, Table 2). Further, the requirement of remifentanil and Dex were significantly lower in the RD2 group than in the RD1 group (*P* < 0.05, Table 2). The duration of surgery, anesthesia, estimated blood loss, urine output, infusion volume, and cisatracurium dosage did not differ significantly among the three groups (*P* > 0.05, Table 2). The number of patients needing vasoactive agents during surgery was significantly less in both the RD1 and RD2 groups (*P* < 0.05, Table 2). Additionally, the recovery time was significantly shorter only in the RD2 group (*P* < 0.05, Table 2). The dermatomal distribution along the midclavicular line of the blocked side was comparable among the three groups (*P* > 0.05, Table 2).

**Table 2:** Intraoperative variables.

	**Group R (***n*** = 36)**	**Group RD1 (***n*** = 36)**	**Group RD2 (***n*** = 36)**	***P*** **-values**
Duration of surgery (min)	125.74 ± 18.78	136.02 ± 23.03	129.04 ± 21.45	0.114
Duration of anesthesia (min)	159.61 ± 20.81	167.35 ± 27.46	165.72 ± 23.52	0.360
Intraoperative bleeding (ml)	105.61 ± 30.45	98.23 ± 21.99	112.03 ± 30.34	0.115
Fluids (ml)	1537.59 ± 203.82	1625.09 ± 178.35	1668.35 ± 216.66	0.062
Urine output (ml)	557.12 ± 62.29	529.03 ± 53.16	537.72 ± 45.25	0.083
Dexmedetomidine (μg·kg^−1^·h^−1^)	0.42 ± 0.13	0.34 ± 0.11*	0.25 ± 0.04^*#^	0.001
Remifentanil (μg·kg^−1^·min^−1^)	0.16 ± 0.06	0.11 ± 0.06*	0.08 ± 0.04^*#^	0.007
Propofol (μg·ml^−1^)	3.56 ± 0.35	2.59 ± 0.18*	2.52 ± 0.12*	0.001
Cisatracurium dosage (mg·kg^−1^)	0.37 ± 0.06	0.34 ± 0.05	0.35 ± 0.05	0.058
Recovery time (min)	18.78 ± 6.46	19.95 ± 7.88	15.02 ± 4.73^*#^	0.005
Number of using vasoactive agent, n (%)	23 (63.89%)	12 (33.33%)*	13 (36.11%)*	0.020
Dermatomal distribution	T5 (T3–T9)	T5 (T2–T8)	T5 (T2–T9)	0.893

*Variables presented as mean ± SD, number of patients n (%) or median (interquartile range)*.

* *P < 0.05 vs. Group R*,

# *P < 0.05 vs. Group DR1*.

### Post-operative Variables

**Figure 3:**
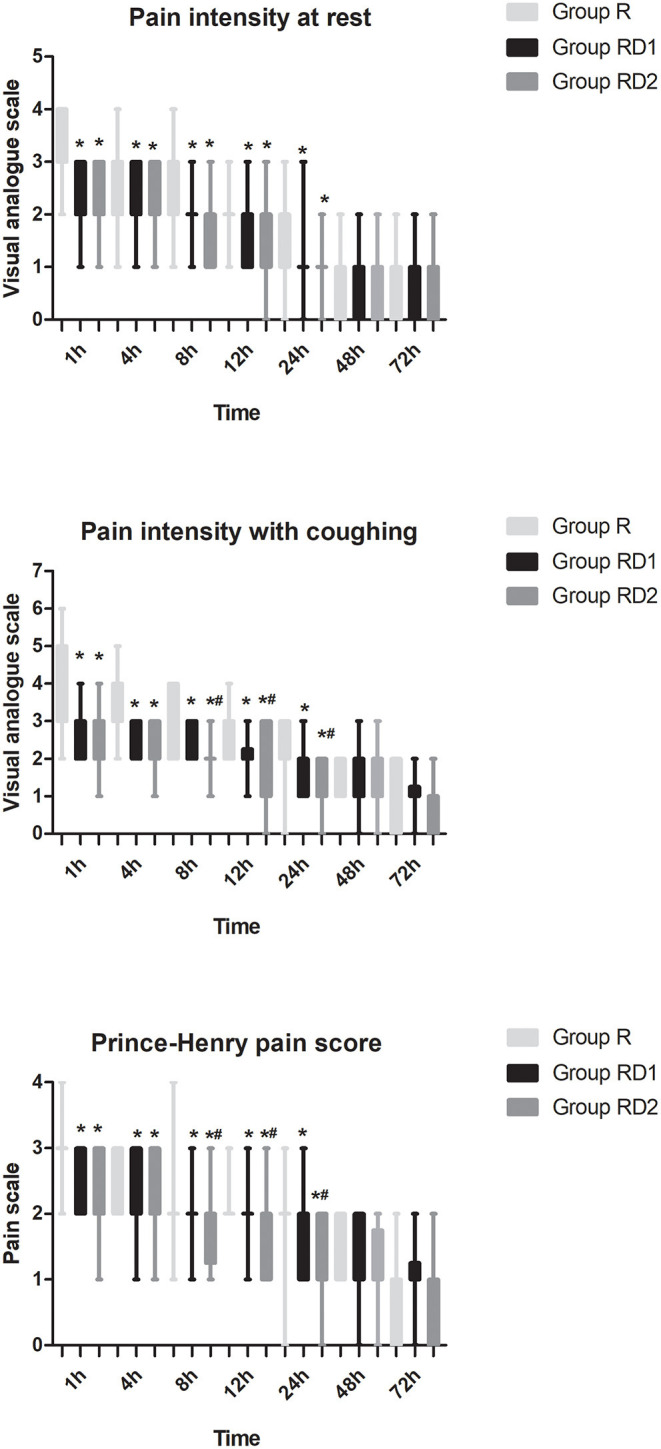
Post-operative pain intensity (at rest and with coughing and Prince Henry pain score) during the first 72 h after surgery among the three groups. *P < 0.05 vs. group R, ^#^*P* < 0.05 vs. group RD1.

**Figure 4:**
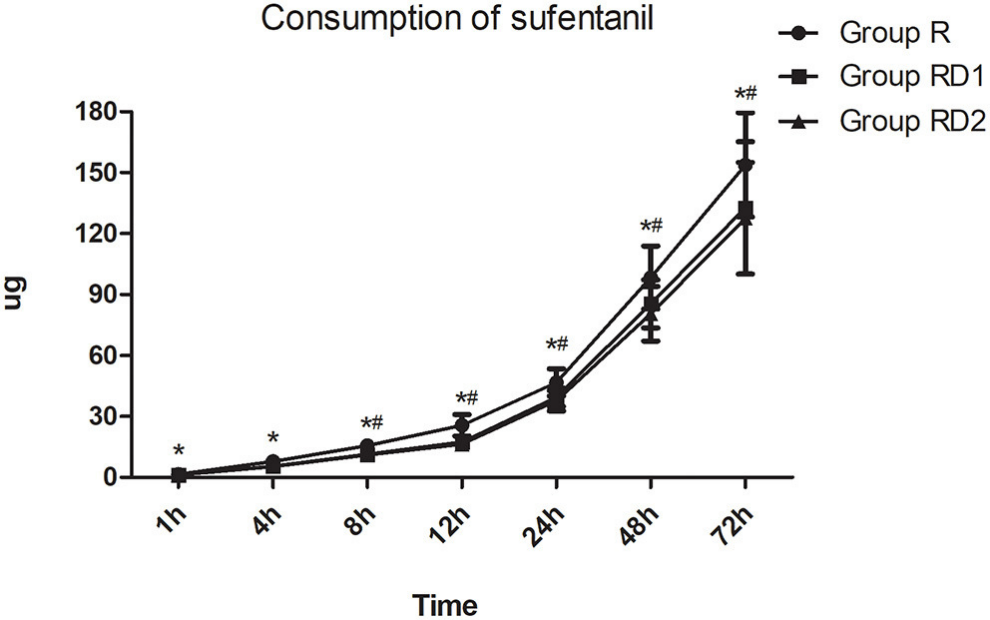
Post-operative sufentanil requirement during the first 72 h after surgery among the three groups. *P < 0.05 vs. group R, ^#^*P* < 0.05 vs. group RD1.

The VAS both at rest and with coughing and the Prince Henry pain score were significantly lower in both the RD1 and RD2 groups than in the R group during the first 24 h after surgery (*P* < 0.05, Figure 3). Furthermore, both VAS with coughing and the Prince Henry pain score were significantly lower in the RD2 group than the RD1 group 8–24 h after surgery (*P* < 0.05, Figure 3). The requirement of sufentanil during the first 72 h after surgery was significantly lower in the RD2 group than the R and RD1 groups (*P* < 0.05, Figure 4).

The total number of press times of PCIA was significantly lower during the first 72 h after surgery in both the RD1 and RD2 groups than the R group (*P* < 0.05, Table 3). Less rescue medication was used in the RD2 group in the post-operative 72 h (*P* < 0.05, Table 3). The time to the first dose of rescue ketorolac was significantly longer in both the RD1 and RD2 groups than in the R group (*P* < 0.05, Table 3). Between the RD1 and RD2 groups, the time to the first dose of rescue ketorolac was significantly longer in the latter (*P* < 0.05, Table 3). Moreover, the satisfaction scores of the patients and surgeons were significantly higher in both the RD1 and RD2 groups than the R group (*P* < 0.05, Table 3). Compared with the R and RD1 groups, time to anal exhaust, removal of chest tubes, and ambulation were significantly shorter in the RD2 group (*P* < 0.05, Table 3). There were no significant differences concerning hemodynamics (from T7 to T9) and time until the first feeding among the three groups (*P* > 0.05, Figure 2, Table 3).

**Table 3:** Post-operative variables.

	**Group R (***n*** = 36)**	**Group RD1 (***n*** = 36)**	**Group RD2 (***n*** = 36)**	***P*** **-values**
Total press times of PCIA	18.23 (12.24–27.14)	11.08 (8.37–17.69)*	10.73 (7.24–19.25)*	0.032
Patient satisfaction score	7.75 (7.25–9.00)	9.00 (7.75–9.75)*	9.00 (8.25–9.50)*	0.045
Surgeon satisfaction score	8.00 (7.50–8.75)	9.25 (8.50–9.50)*	9.25 (8.75–9.75)*	0.037
Number of rescue medication, n (%)	14 (38.89%)	12 (33.33%)	5 (13.89%)^*#^	0.044
Time to first dose of rescue ketorolac (h)	1.75 (0.47–3.25)	4.69 (3.24–7.42)*	6.46 (3.88–10.23)^*#^	0.013
Post-operative rehabilitation indexes				
Anal exhaust (h)	24.34 (13.56–33.19)	21.57 (12.41–31.78)	17.39 (13.97–25.23)^*#^	0.039
Time until the first feeding (h)	4.74 (4.08–6.17)	4.65 (4.11–5.02)	4.83 (4.16–5.12)	0.375
Removal of chest tubes (d)	2.65 (2.31–3.67)	2.42 (1.98–3.03)	1.95 (1.47–2.98)^*#^	0.048
Ambulation (d)	3.25 (2.42–3.70)	2.74 (2.06–3.31)	2.17 (1.81–3.05)^*#^	0.013

*Variables presented as median (interquartile range) or number of patients n (%). PCIA, patient controlled intravenous analgesia*.

* *P < 0.05 vs. Group R*,

# *P < 0.05 vs. Group RD1*.

The incidence of tachycardia and PONV was significantly reduced in the RD2 group (*P* < 0.05, Table 4). Pneumonia was more frequent in the R group than the other two groups, but this difference was not significant. No patients experienced puncture-related complications and respiratory inhibition during the study. The prevalence of chronic pain was lower and QoR-40 scores higher in the RD2 group than the other two groups 3 months after surgery (*P* < 0.05, Table 4).

**Table 4:** Adverse effects and post-operative recovery.

	**Group R (***n*** = 36)**	**Group RD1 (***n*** = 36)**	**Group RD2 (***n*** = 36)**	***P*** **-values**
Hypotension	5 (13.89%)	3 (8.33%)	4 (11.11%)	0.927
Tachycardia	11 (30.56%)	9 (25.00%)	2 (5.56%)^*#^	0.018
Pruritus	4 (11.11%)	5 (13.89%)	1 (2.78%)	0.335
PONV	18 (50.00%)	15 (41.67%)	6 (16.67%)^*#^	0.008
Pneumonia	8 (22.22%)	3 (8.33%)	2 (5.56%)	0.116
Respiratory inhibition	0	0	0	1.000
Puncture related complications	0	0	0	1.000
Prevalence of chronic pain	14 (38.89%)	6 (16.67%)	5 (13.89%)^*#^	0.038
QoR-40 scores	160 (155–170)	169 (166–172)	181 (177–184)^*#^	0.041

*Variables presented as number of patients n (%) or median (interquartile range)*.

* *P < 0.05 vs. Group R*,

# *P < 0.05 vs. Group RD1*.

## Discussion

This double-blind randomized controlled trial (RCT) showed that 30 ml 0.375% ropivacaine with 0.1 mg/kg dexamethasone plus 1.0 μg/kg DEX for bi-level ESPB provided superior analgesia for patients undergoing VATS, especially during the first 24 h after surgery. The discharge from the PACU; time to the first dose of rescue analgesia; the number of rescue medications; total press times of PCIA; and time to anal exhaust, removal of chest tubes, and ambulation were all lower and the QoR-40 scores higher in the RD2 group. Additionally, the incidence of tachycardia, PONV, and chronic pain were all reduced.

With fewer invasive surgical techniques being performed in thoracic surgery, anesthesiologists need to identify less invasive analgesia regimens (23). A previous study found that delayed mobilization was independent of predictors of delayed discharge (24). The increased awareness of opioid dependence and overuse, opioid-related tolerance, and hyperalgesia has inspired anesthesiologists to take action to minimize the use of perioperative opioids (25). Under ultrasound guidance and with a better understanding of the nervous system anatomy, multimodal and regional analgesia have emerged as the important opioid-sparing techniques. The failure rate of TEB and PVB was up to 15% and may be associated with severe complications. Although intercostal nerve block is highly effective, it requires multiple injections and is time-consuming (26). Hence, more precise dermatomal blocks that can facilitate effective analgesia and minimize the risk of puncturing adjacent structures away from the midline are needed (27). As a result, several thoracic wall blocks ranging from the parasternal to the intercostal plane have been described. These rely on the passive spread of LA to target nerves within the plane or in adjacent tissue. The effect of analgesia is dependent on multiple factors such as the volume of LA and the direction and speed of injection. This results in an inevitable individual variation in the extent and intensity of sensory loss (28, 29). Considering the cardiotoxicity of bupivacaine and the sensory-motor separation of ropivacaine, we decided to use 0.375% ropivacaine in this study.

Erector spinae plane block was first described by Forero et al. in 2016 for both post-thoracotomy neuropathic and acute postsurgical pain. The authors proposed that the dorsal and ventral rami of the thoracic spinal nerves were the ESPB action sites, which also produced a multidermatomal sensory block (11). A subsequent study reported that when ESPB was performed at the level of the T5 vertebra, LA could spread between T3 and L2 (30). Briefly, the 20 ml LA administered in the ESPB at the T5 level was shown to spread to five levels in the intercostal plane, while the neuronal foraminal and epidural spread was only limited to 2–3 levels in one cadaver study (31). However, another cadaver study stated that it expanded to the outer surface of the thoracic wall with no spread to the paravertebral space and minimal spread to the dorsal ramus (32). The analgesic duration of ESPB in the RD2 group was longer than that reported in a previous study (27). This is likely because of the different comprehensive effects of pre-emptive and multimodal analgesia in our study, especially due to the opioid-sparing mechanisms of DEX which include centrally mediated analgesia, action on peripheral nerve α_2_B-adrenoceptors, and attenuation of the inflammatory response (33). Besides, we used smaller incisions and improved muscle-sparing techniques as described in a previous study (17). It must be noted that the pattern of sensory innervation is complex and non-segmental because of the communication and anastomosis between adjacent spinal nerves and their branches in the chest (34). As a result, ESPB can only be used as part of a multimodal analgesia regimen or as a valid alternative to conventional regional techniques, especially for high-risk patients such as full heparinization for perioperative pain management in cardiac surgery although research about this area is still in its infancy (14, 35).

Previous studies have shown the analgesic effect of using a single-shot ESPB after breast surgery, rib fractures, and laparoscopic cholecystectomy (36–38). Additionally, Gaballah KM et al. reported that single-shot ESBP provided superior analgesia and longer time to the first required analgesia than serratus anterior plane block (SAPB), which is another recent analgesia technique done by blocking the intercostal nerves and their lateral cutaneous branches (39). However, we still adopted the bi-level ESPB technique based on a previous study that reported that bi-level ESPB was better than a single, large-volume injection in reducing opioid rescue (13). Our study showed lower VAS both at rest and with coughing and lower Prince Henry pain scores than those reported in a previous study (21). This is probably because ESPB could provide both somatic and visceral analgesia, particularly at early post-operative time points (36). Another previous study reported that rebound pain is a very severe type of pain that appears when the peripheral nerve block wears off (40). We also observed this phenomenon in some patients despite adopting the multimodal analgesia regime in our study.

Twenty-three patients in the R group were treated with a vasoactive agent during the operation, while only 12 and 13 patients in the RD1 and RD2 groups, respectively, needed the vasoactive agent. We considered that the higher number of using vasoactive agents may be a result of the lesser consumption of general anesthetics in the RD1 and RD2 groups. In this study, there was no significant difference in the post-operative feeding time among the three groups. However, the overall time was significantly shorter than that reported in a previous study (41). The time for anal exhaust, removal of chest tubes, and ambulation were reduced in the RD2 group, which may be because of the significantly reduced need for sufentanil and appropriate control of post-operative pain.

Ultrasound-guided ESPB may be relatively simpler and safer than conventional standard techniques because transverse processes offer convenient sonographic landmarks and act as an anatomical barrier to avoid needle insertion into the pleura. Because the targets for injection are distant from the midline of the spine, there is little risk of spinal cord or nerve injury, epidural hematoma or infection, major vascular injury, pleural puncture, and lung injury (42). Consistent with a previous study, we also did not record any puncture-related complications in our study, which may be because too few patients were recruited to adequately detect differences (22). The numbers of patients with tachycardia were significantly reduced in the RD2 group, demonstrating the stability of the cardiovascular system which is probably due to the combination with higher dosages of DEX (16). A previous study showed that the total plasma levobupivacaine concentrations in the ESPB group were significantly lower than those in the TPVB group even with the continuous infusion of 0.2% levobupivacaine (8 ml/h). However, no patients showed LA systemic toxicity (LAST) (43). As a result, we did not measure the plasma ropivacaine concentration in this study. Although there are no reported published cases of LAST after thoracic wall blocks, the toxic plasma concentration of LA is still considered to be established (27). Besides, the following precautions should be taken routinely: within minimum recommended weight-based LA limits and availability of the LAST rescue kit (44). A previous study has reported that ESPB could partially block C7–C8 including VATS (30). Therefore, future studies should explore the untoward effects of blocking the lower cervical dermatomes.

Chronic post-surgical pain is defined as recurring or persisting pain for more than 2 months after surgery. The reported incidence of chronic postsurgical pain (CPSP) is between 20 and 60% among VATS (45). Erector spinae plane block with 25 mg of levobupivacaine and 40 mg of triamcinolone has been used safely and effectively to treat CPSP in a case series of seven patients undergoing different surgical procedures. The reason for this is perhaps the local corticosteroid dose could suppress a continuous inflammatory condition and ectopic discharge in neural membranes (46). Therefore, we added 0.1 mg/kg of dexamethasone during the ESPB in each group in this study. The patients in the RD2 group demonstrated higher QoR-40 scores which proved better long-term post-operative recovery than for patients in the R and RD1 groups.

Our study has some limitations. First, this was a single-center study. More patients from different centers should be included to test the reproducibility of our results. Second, we only explored 30 ml 0.375% ropivacaine with two different doses of DEX in this study; the optimal dosing regimen of different combinations of drugs for ESPB needs to be further explored. Besides, we did not compare the analgesic effect between the ultrasound-guided continuous and bi-level ESPB for technical and economic reasons (47). Third, several factors influenced the outcome of the study such as the cultural level, behavior of smoking, and drinking. Fourth, VATS can greatly shorten the length of hospital stay and reduce the cost of hospitalization. However, we did not record these indicators though the time to anal exhaust, removal of chest tubes, and ambulation were significantly shorter in this study (7). Fifth, the removal of chest tubes and ambulation initiated by the caregivers could be biased although they have received consistent professional training and obtained corresponding qualifications. Last, we adopted the Chinese version of the QoR-40 questionnaire in this study. Cultural differences may have biased the results of this study (48).

In summary, performing a pre-emptive ultrasound-guided bi-level ESPB plus 1 μg/kg DEX provided superior analgesic effects and long-term post-operative recovery for patients undergoing VATS, while adverse effects were also reduced. More randomized controlled trials are needed to explore the efficacy and safety of ultrasound-guided bi-level ESPB with different combinations of drugs for patients undergoing VATS.

## Data Availability Statement

The raw data supporting the conclusions of this article will be made available by the authors, without undue reservation.

## Ethics Statement

The studies involving human participants were reviewed and approved by the Institutional Review Board of Liaocheng People's Hospital provided Ethics Approval to conduct the trial. The patients/participants provided their written informed consent to participate in this study.

## Author Contributions

All authors listed have made a substantial, direct, and intellectual contribution to the work and approved it for publication.

## Conflict of Interest

The authors declare that the research was conducted in the absence of any commercial or financial relationships that could be construed as a potential conflict of interest.

## Publisher's Note

All claims expressed in this article are solely those of the authors and do not necessarily represent those of their affiliated organizations, or those of the publisher, the editors and the reviewers. Any product that may be evaluated in this article, or claim that may be made by its manufacturer, is not guaranteed or endorsed by the publisher.
